# Medicine

**Published:** 1894-12

**Authors:** R. Shingleton Smith


### Progress of the Medical Sciences.

### MEDICINE.

We have recently had a very important paper on the treatment of pneumonia from Dr. D. J. Leech.[1] He deals with the question, how far the teaching of experience may be trusted, or whether we should not be guided by our knowledge of the pharmacological influence of remedial agents in the conditions which are present. After an analysis of the statistics of general hospitals, of the Army Medical Reports, and of the records of individual experiences of practitioners, he points out that none of these statistics prove the value of any one treatment, and that remedies very opposite in character may under different conditions do good. The tendencies of the ailment manifestly vary with time and place; and these tendencies, as well as the symptoms present, should be carefully noted, and should guide our treatment. He concludes as follows: "Finally, it is evident that exactness, full details, and a record of associated circumstances are necessary to make pneumonia statistics useful. The value of the older data is greatly diminished by their bareness; but it was impossible, in former days to give that information which is now easy to record, and which there is good hope will accumulate and lead us to more definite views concerning treatment. It may, indeed, be impossible to show by figures the precise advantages and disadvantages of every method of treatment; but we may surely hope that with the aid of increased knowledge as to the effects of remedies which exact observation must give us, the time will come when we shall not have to report a hospital death-rate from pneumonia of one in 3.2 to 3.9 in those over fifteen years of age."

Of the individual methods of treatment analysed, the most noteworthy of the modern modes is that advocated for ten or twelve years past by Petresco, of Bucharest, who professes to "jugulate" pneumonia by large doses of digitalis. The results he gives are more favourable than any hitherto recorded, and show that, under the special circumstances mentioned—viz., among young soldiers at Bucharest,—deaths are extremely rare when digitalis is given in enormous doses. In 1891 he had treated 825 cases, with a mortality of 2.06 per cent.,[2] and in 1893 he claimed to have treated successfully 1,061 cases during the last twelve years.[3] He gives one to three drachms daily of digitalis leaf in infusion (ʒj. in ℥viiss.), a table-

[1] *Med. Chron.*, Sept., Oct., 1894. [2] *Therap. Monatsh.*, Feb., 1891.

[3] *Rev. de Méd.*, Mar. 10, 1893.

spoonful (nearly four grains) being given every half-hour. The strength of our British infusion is twenty-eight grains to ten ounces, so that the drug is rarely given in doses approaching those which are found by Petresco to jugulate the malady in three days, fever and physical signs alike disappearing as by enchantment. Numerous observations by other physicians tend to corroborate results which, according to Dr. Leech, are, and pending further information must remain, a puzzle to us; but the specific curative influence claimed by Petresco is not accepted, although there is a general consensus of opinion that digitalis is more or less useful in counteracting the tendency to heart failure, and that it may safely be given in doses far in excess of those admissible in cases of valvular disease. This question has long been *sub judice*, and seems likely to continue so until the details given by Petresco are faithfully carried out: where he has led the way, careful observers in hospitals need have no fear to follow.

I have often been astonished to find how little effect digitalis, in what are commonly considered to be full doses, appears to have in modifying the natural history of pneumonia, and I have never had the good fortune to see anything which could be spoken of as "jugulation," although half-ounce doses of the English infusion have been given every hour for the whole twenty-four hours. It cannot be difficult to decide the question which Petresco has put so definitely before us; if his statements are confirmed by other observers under varying surrounding conditions, we should hear no more of the folly of trusting to Nature in the treatment of so fatal a malady as pneumonia has hitherto proved itself to be.

Functional conditions of the heart are attracting much attention, and in connection therewith the subject of thyroid feeding is of importance.

The results of the treatment of myxœdema by thyroid feeding have been already summarised in this *Journal*.[1] This has now long passed the experimental stage and has become a matter of daily routine; but the experimental process has gone astray in various directions, and we find that thyroid preparations are being given in a variety of maladies without rhyme or reason. It is scarcely possible just now to read through a medical journal without finding some reference to the use of thyroid extracts or tabloids in cases of exophthalmic goitre; and it is inferred that because one case of this kind has improved under observation, that the treatment is not only justifiable but has proved itself to be useful. Theoretical considerations would tend to indicate that in cases of thyroid enlargement the use of thyroid preparations should be carefully avoided, and the fact that these cases are not always made worse but sometimes get

[1] Vol. xi., 1893, pp. 32, 255.

better in spite of the treatment tends to show how much the phenomena of Graves's disease are of the functional and temporary character. Various papers on exophthalmic goitre, more particularly those of Arthur Maude, have thrown fresh light on the pathology of the malady, and tend to indicate that a general implication of the nerve centres by some such toxine as is produced by the enlarged thyroid is probably the cause of the diffused and varied phenomena of the disease; that, in fact, the thyroid is probably the *fons et origo mali*, and is the organ to which curative rather than palliative measures of treatment should be directed. He concludes one of his papers as follows: "Pathologists have long sought to explain the phenomena of this disease by some focal lesion in the sympathetic or the medulla. But as our clinical knowledge grows, the more unsatisfactory such explanations become. The old sympathetic theory died hard—killed by Filehne and Dr. Hale White; but will any lesion in the medulla do more than explain the cardio-vascular symptoms? I think not. We must regard the neurosis as a nerve poisoning for the present: the brunt undoubtedly falls on the medulla, but it is felt from the cortex to the periphery. What that poison is we do not know. That there are probably secondary poisons, I have indicated. But remember the one feature, so peculiar to the disease, the thyroid change. To my mind, the universal goitre, the connection with myxœdema, the results of operations on the thyroid, point to that gland as the fountain-head of evil."[1]

There is a widely-spread and growing belief that the neural and cardiac phenomena are due to the explanation given by Greenfield and Maude, that of a production (or non-elimination) in the thyroid itself of some toxine, which acts on the whole nervous system even to the peripheral nerves, though the brunt of its action falls on the vaso-motor centres of the medulla and some neighbouring centres. The question of the exact *rôle* played by the thyroid change is difficult; it is clear that all morbid changes in the thyroid are not operative to produce Graves's disease, but all the cases (in which the gland has been properly examined) show two common factors: cell proliferation and diminution of colloid.[2]

This question was fully considered by Dr. Greenfield in his Bradshaw lecture,[3] and the antithesis of the clinical phenomena of myxœdema and Graves's disease was there commented upon.

The tachycardia of Graves's disease, being due to the presence of a toxine produced by the thyroid, naturally suggests the question whether other forms of functional heart-disturbance may be due to similar causes. The discussion on functional diseases of the heart, introduced by Dr. Douglas Powell, at the annual meeting of the British Medical Association in Bristol, did not elicit any further facts in this direction; even in cases

[1] *Tr. M. Soc. Lond.*, vol. xvii. [2] *Brain*, 1894.

[3] *Brit. M. J.*, 1893, vol. ii., p. 1261.

of paroxysmal tachycardia, the neurosal origin of the condition known as "runaway heart" was the only one suggested, but it is difficult to dissociate cases of this kind from the slighter forms of Graves's disease.

The discussion commenced with a very complete summary by Dr. R. Douglas Powell, who gave an excellent outline of the varieties of cardio-vascular hyperæsthesia, and of paroxysmal and sustained heart acceleration and retardation; and this was followed by a paper on the irregular heart of influenza, by Dr. A. E. Sansom. It appears to be generally admitted that in the treatment of the irregularity, as well as of the tachycardia, all cardiac tonics are singularly inefficacious. Digitalis has proved not only useless, but harmful. Belladonna seems to have given somewhat better results. Dr. Sansom has found the galvanic current to the vagus to be the most effectual method of controlling irregularity; but he admits that there is seldom much amendment under six months, even when the applications are made for six minutes three times a day. We fear that most medical men would tire of employing so slow a therapeutic agent as this, and most patients would be weary both of it and the doctor long before the beneficial effect could become obvious. My own attempts at galvanisation of the vagus or sympathetic (or as it most commonly must be, both simultaneously, or neither) have not given much encouragement to persevere in a course which seemed to be altogether ineffectual.

The demonstration by Dr. Frank J. Wethered of the treatment of chronic diseases of the heart by baths and gymnastics, as practised by the brothers Schott at Nauheim, opens up a far more promising field of heart therapeutics. The "terraincur" of Oertel has shown itself to be of use, although the system of graduated exercises in place of rest has not been generally accepted by the profession in this country. Schott's plan of giving exercises, or "movements with resistance," has, according to Dr. Wethered, given astonishing results. Each exercise is made extremely slowly and regularly, and is resisted by the doctor or trained attendant, who carefully regulates the degree of resistance in accordance with the condition of the patient.

The effect of this method of treatment has been marked: the pulse becomes more regular and its volume increases, the rate is also diminished, and the area of cardiac dulness after half-an-hour's exercise is found to be diminished to an astonishing degree. These are facts dependent simply on the observation of the attendant; but the improvement is also marked in the general condition and reports of the patients themselves. Dr. Schott is of opinion that all forms of heart disease may derive benefit from this treatment, with the exception of cases of aneurism and those in which there is an advanced degree of arterio-capillary fibrosis with high tension; and in Dr. Wethered's opinion the benefit has not been overestimated.[1]

[1] *Brit. M. J.*, 1894, vol. ii., p. 1045.

The following tabular recapitulation of the treatment of "the senile heart," given by Dr. G. W. Balfour, is likely to be of service[1]:—

In every case careful removal of *lædentia*.

*Precordial Anxiety.*—Careful dieting; cardiac tonics; rest at first, afterwards regulated exercise.

*Intermission and Irregularity.*—Careful dieting; vascular stimulants, combined with cardiac tonics; sedatives, especially for women about their climacteric, occasionally hypnotics; antacids and anti-arthritics; assafœtida (pil. galbani co.); moderate exercise.

*Palpitation.*—Antacids; stimulants; mustard over precordial region; hot foot-baths. In interval strengthen patient by open-air exercise, good food, and such tonics as may seem needful, especially iron.

*Tremor Cordis.*—Careful dieting most important; antacids; anti-arthritics; *pil. galbani co.*

*Tachycardia.*—Careful dieting; in recent cases following cardiac overstrain, belladonna, or atropine, must be pushed till pupils dilate. In cases of poisoning by tobacco or alcohol, tonic doses of digitalis useful. Cardiac tonics, especially digitalis and arsenic, continued for a long time in moderate doses, supplemented by hypnotics at bedtime, especially morphia. Digitalis most useful in vagus paralysis, morphia in affections of the sympathetic. Cholate of soda slows the pulse, but it destroys the blood corpuscles, and the benefit is thus a doubtful one. Antipyrine has been recommended theoretically. Faradization of the skin over the *precordia*, or of the vagus nerve; or the skin or vagus may be galvanized. Compression of the vagus. Forced inspiration, holding the breath as long as possible. Ether sprayed along the cervical spine. A chloroform poultice over the precordial region.

*Bradycardia.*—In the hemi-systolic variety, cardiac tonics, especially digitalis. In true bradycardia digitalis is also indispensable, to maintain the elastic tonicity of the heart, and to enable the heart to cope with the exceptionally high blood pressure prevalent during part of the systole.

*Delirium Cordis.*—Careful dieting, vascular stimulants, cardiac tonics, antacids, and anti-arthritics.

*Angina Pectoris.*—During the paroxysm, nitro-glycerine, nitrite of amyl, chloroform and morphia. During the interval most careful and abstemious diet, especially towards evening. Vascular stimulants in combination with cardiac tonics, especially arsenic. Exercise is to be avoided, and only undertaken when duly prepared for by the ingestion of some vascular stimulant.

R. SHINGLETON SMITH.

[1] *The Senile Heart*, 1894, p. 292

